# Tracing temporal and geographic distribution of resistance to pyrethroids in the arboviral vector *Aedes albopictus*

**DOI:** 10.1371/journal.pntd.0008350

**Published:** 2020-06-22

**Authors:** Alessandra Tancredi, Davide Papandrea, Michele Marconcini, Rebeca Carballar-Lejarazu, Mauricio Casas-Martinez, Eugenia Lo, Xiao-Guang Chen, Anna R. Malacrida, Mariangela Bonizzoni

**Affiliations:** 1 Department of Biology and Biotechnology, University of Pavia, Pavia, Lombardy, Italy; 2 Centro Regional de Investigación en Salud Pública, Instituto Nacional de Salud Pública, Tapachula, Chiapas, Mexico; 3 Department of Biological Sciences, University of North Carolina, Charlotte, North Carolina, United States of America; 4 Department of Pathogen Biology, School of Public Health, Southern Medical University of Guangzhou, China; USDA-ARS Center for Medical Agricultural and Veterinary Entomology, UNITED STATES

## Abstract

**Background:**

The arboviral vector *Aedes albopictus* became established on all continents except Antarctica in the past 50 years. A consequence of its rapid global invasion is the transmission of diseases previously confined to the tropics and subtropics occurring in temperate regions of the world, including the re-emergence of chikungunya and dengue in Europe. Application of pyrethroids is among the most widely-used interventions for vector control, especially in the presence of an arboviral outbreak. Studies are emerging that reveal phenotypic resistance and monitor mutations at the target site, the *para* sodium channel gene, primarily on a local scale.

**Methods:**

A total of 512 *Ae*. *albopictus* mosquitoes from twelve geographic sites, including those from the native home range and invaded areas, were sampled between 2011 and 2018, and were analyzed at five codons of the *para* sodium channel gene with mutations predictive of resistance phenotype. Additionally, to test for the origin of unique *kdr* mutations in Mexico, we analyzed the genetic connectivity of southern Mexico mosquitoes with mosquitoes from home range, the Reunion Island, America and Europe.

**Results:**

We detected mutations at all tested positions of the *para* sodium channel gene, with heterozygotes predominating and rare instance of double mutants. We observed an increase in the distribution and frequency of F1534C/L/S mutations in the ancestral China population and populations in the Mediterranean Greece, the appearance of the V1016G/I mutations as early as 2011 in Italy and mutations at position 410 and 989 in Mexico. The analyses of the distribution pattern of *kdr* alleles and haplotype network analyses showed evidence for multiple origins of all *kdr* mutations.

**Conclusions:**

Here we provide the most-up-to-date survey on the geographic and temporal distribution of pyrethroid-predictive mutations in *Ae*. *albopictus* by combining *kdr* genotyping on current and historical samples with published data. While we confirm low levels of pyrethroid resistance in most analyzed samples, we find increasing frequencies of F1534C/S and V1016G in China and Greece or Italy, respectively. The observed patterns of *kdr* allele distribution support the hypothesis that on site emergence of resistance has contributed more than spread of resistance through mosquito migration/invasions to the current widespread of *kdr* alleles, emphasizing the importance of local surveillance programs and resistance management.

## Author summary

*Aedes albopictus* is a highly invasive mosquito and a vector for a number of arboviruses. The arrival and establishment of *Ae*. *albopictus* in temperature regions of the world, such as Europe, has been accompanied by re-emergence of arboviral diseases. There are no effective therapeutic treatments for arboviruses meaning control of vector populations is the primary strategy to prevent arboviral disease transmission. Pyrethroids are frequently used for control of vectors based on their low mammalian toxicity and rapid knockdown effect on mosquitoes. The identification of mutations predictive of resistance phenotype in the *para* sodium channel gene, the target site of pyrethroids, has provided for molecular markers to test for resistance by genotyping wild-collected mosquitoes. Here we analysed all currently known predictive mutations for pyrethroid resistance in 512 geographic mosquitoes sampled in a span of seven years. Thus, we are able to add a temporal dimension to the analysis of geographic distribution of resistance alleles. Overall our data confirm a patchy distribution of mutations predictive of the resistance phenotype, but also reveal an alarming increase of resistance mutations in China, Greece and Italy. Resistance mutations appear to have arisen locally more than being spread through mosquito migration/invasions.

## Introduction

Urbanization, globalization and increased international mobility have made the vector-borne viral diseases, dengue, Zika and chikungunya fever, threats to a large fraction of the human population [1]. Historically, dengue and chikungunya cases were confined to tropical and sub-tropical regions of the world, but recent years have seen their expansion into temperate areas [2]. For instance, autochthonous cases of dengue and chikungunya have been reported in southern France and Croatia since 2010 and Italy suffered from chikungunya outbreaks in 2007 and 2017 [3–7]. The re-emergence of dengue and chikungunya in Europe are dependent on the arrival and establishment of their competent vector, the mosquito *Aedes albopictus* [8,9]. *Aedes albopictus* is an aggressive invasive species that moved out of its native home range in Southeast Asia reaching every continent except Antarctica in the past 40–50 years [9]. The rapid worldwide spread of *Ae*. *albopictus* was human-mediated and occurred through the passive movements of propagules resulting in lack of isolation by distance and in genetic admixture of new populations [10–14].

Personal protection measures and vector control operations are critical to prevent disease transmission because there are limited vaccines available and no specific therapeutic treatments against dengue, Zika and chikungunya infections. The use of chemical compounds, mainly through fogging, is an important element of vector control against *Ae*. *albopictus* populations [15,16]. Pyrethroids (PY) are the most commonly used compounds because of their low mammalian toxicity and rapid mosquito knockdown [16]. Extensive use of insecticides for the control of *Ae*. *albopictus* and, in some regions, other sympatric vectors (i.e. *Aedes aegypti*, *Culex quinquefasciatus* and anopheline vectors) imposes selection pressure for resistance. Phenotypic resistance to PYs has been documented since the early ‘2000s in *Ae*. *albopictus* populations from native Asian countries including China, India, Pakistan, Malaysia and Thailand and is emerging in newly-invaded regions such as Africa (e.g. Cameroon, Central African Republic), Europe (e.g. Italy) and the USA (e.g. New Jersey) [17–24]. The identification of phenotypic resistance to PYs is a major concern for the sustainability of current insecticide-based control programs and calls for continuous monitoring [25,26].

In mosquitoes, resistance to PY is physiologically mediated by mutations (knockdown resistance or *kdr* mutations) in the target site, the voltage-gated sodium channel gene (*vgsc*); increased detoxification primarily via cytochrome P450 monooxygenases, glutathione-S-transferase and esterases and altered cuticule [20,27,28]. A small number of amino acid residues are mutated consistently in *vgsc* across a wide range of mosquito species studied in relation to PY resistance, suggesting convergent evolution [29]. As a consequence, these mutations have become the most used and reliable molecular maker for PY resistance in mosquitoes [29]. Positions of *kdr* mutations are numbered according to the house fly sodium channel protein, where the first *kdr* mutation, L1014F, was detected [30]. In *Aedes spp*. mosquitoes, mutations in the *vgsc* gene predictive of the resistance phenotype were not found at position 1014, but at the nearby 1016 position [20]. A total of six mutations at five sites (i.e. V410L, S989P, I1011M, V1016G/I and F1534C) have been further characterized in *Ae*. *aegypti* populations as predictive of the resistant phenotype, each occurring at varying frequency and geographic distribution [20,31–32]. Interestingly, retrospective studies on Mexican mosquitoes showed a sequential evolution of *kdr* mutations: the F1534C mutation, which confers low levels of resistance, emerged first; while the first-studied V1016I mutation evolved on haplotypes carrying the F1534C mutation, with the double mutant rapidly increasing in frequency because it engenders higher resistance [32,33]. A third mutation V410L, which was only recently-identified as associated with resistance, emerged in 2002 and co-evolved with V1016I and F1534C [34]. Importantly, changes in the frequency of *kdr* mutations were detected in *Ae*. *aegypti* mosquitoes collected within spans of less than five-years [32–34].

In a first survey on *Ae*. *albopictus* collected between 2011 and 2014, we found three nonsynonymous changes (F to C, L or S) at position 1534 in populations from China and USA with the mutation to S being the most predictive of the resistance phenotype [35]. Recently, F1534C was reported at low frequency in populations from Brazil, Vietnam and Singapore [36,37]. A mutation to G at position 1016 was detected for the first time in populations from Italy and Vietnam in 2016 and it is strongly predictive of the resistant phenotype [37,38]. The V1016G mutation was later confirmed in Chinese mosquitoes, primarily collected in urban areas of the Beijing municipality [39]. Despite the recognized importance of the quick and continuous dispersal of *Ae*. *albopictus*, current analyses of *kdr* mutations are mainly local and lack both the global and temporal perspectives that are necessary to estimate the efficacy of control interventions based on PYs.

In this study, we asked the following questions: 1) what is the allele frequency distribution pattern of currently-known PY-resistance predictive mutations in native, established and invasive populations of *Ae*. *albopictus*? 2) do we see temporal changes in the distribution of *kdr* haplotypes? 3) did *kdr* mutations arise once and then spread, or have they evolved independently in different populations?

We detected mutations at all currently known PY-resistance predictive sites and identified additional allelic variants; we observed primarily heterozygotes with few instances of the double mutant S989Y-F1534L in China and Mexico. Overall, we observed an increase in the distribution and frequency of mutations at position 1534 and the appearance of mutations at position 1016 as early as 2011 in Italy. Interestingly, mutations at position 410 were the most geographically widespread being detected in mosquitoes from Thailand, Mexico and Italy. Finally, haplotype analyses showed evidence for multiple origins of all *kdr* mutations. Our results underscore the importance of coupling temporal and geographic analyses and imply that resistance to PY is losing its patchy worldwide distribution and emerged earlier than previously thought in invasive *Ae*. *albopictus* populations [35,38]. These results are fundamental to guide the set-up of national vector control programs and have important practical implications for the management and sustainability of PY-based control measures against *Ae*. *albopictus*.

## Results

### Geographic distribution of *kdr* mutations in *Aedes albopictus*

**Fig 1:**
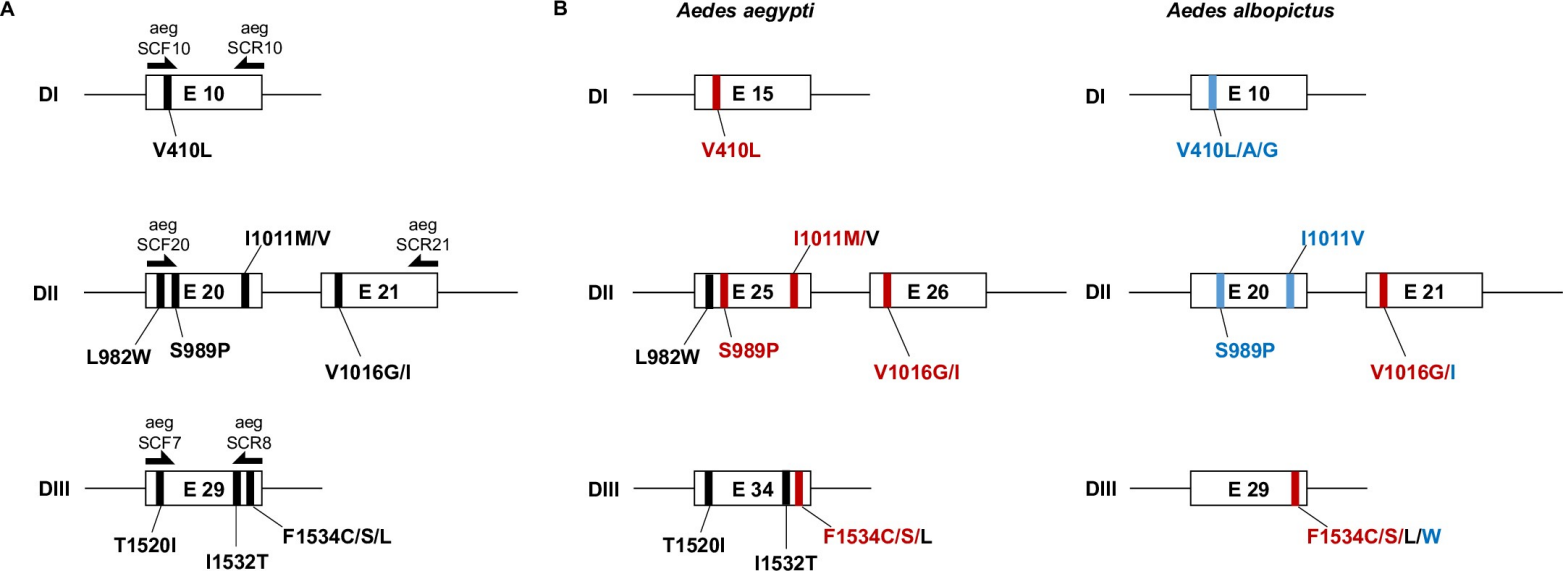
*Kdr* mutations in *Aedes* spp mosquitoes. A) Schematic representation of targeted codons within the *vgsc* gene and used primers; B) *kdr* mutations in *Ae*. *aegypti* and *Ae*. *albopictus* predictive of the resistant phenotype (in red) or novel mutations found in this study (in blue).

**Fig 2:**
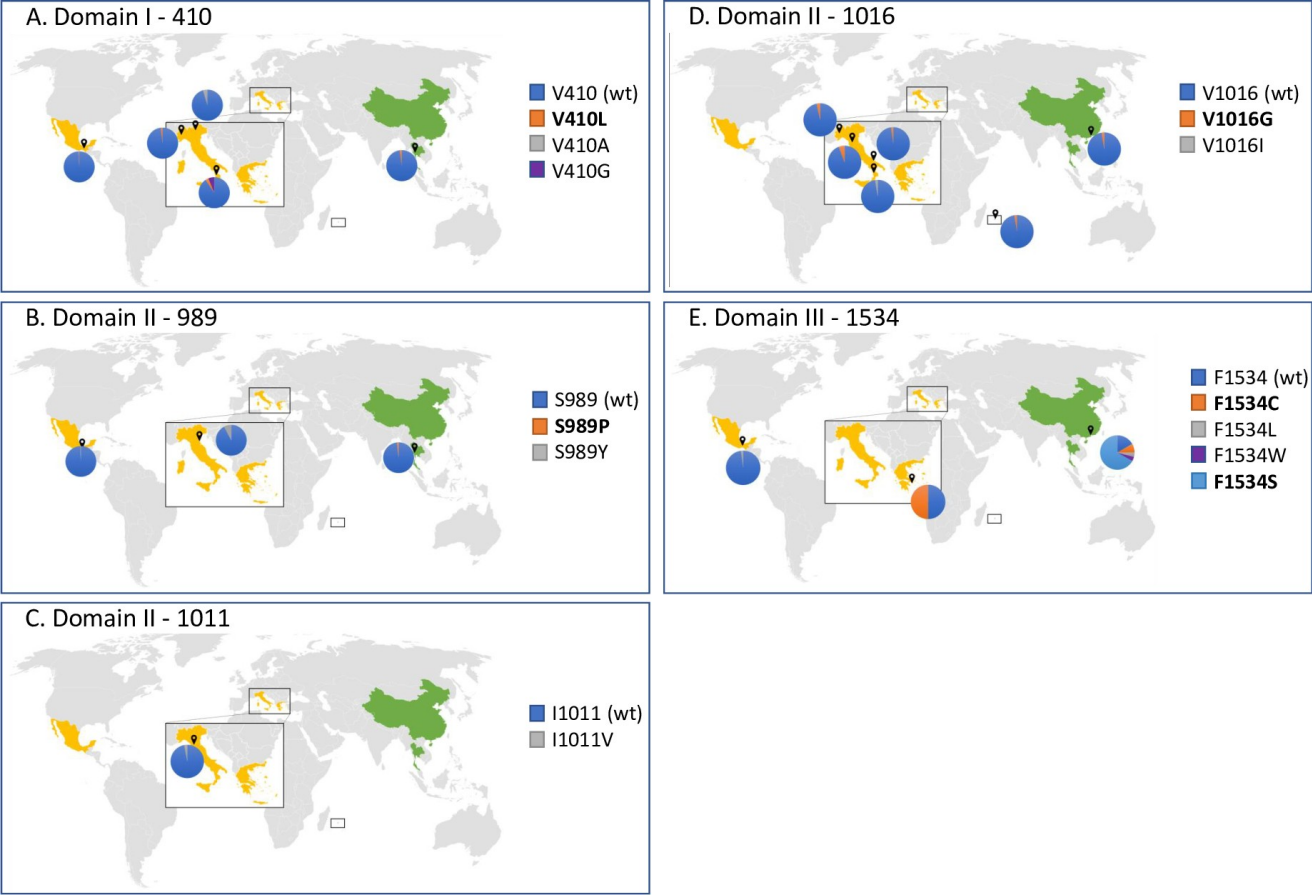
Distribution of PY-predictive mutations in *Ae*. *albopictus* world-wide samples. Frequency distribution of mutations at position 410 (A), 989 (B), 1011 (C), 1016 (D) and 1534 (E) of the para-sodium channel gene of *Ae*. *albopictus*. Only samples in which *kdr* mutations were identified are shown. Invasive and native populations are labelled in yellow and green, respectively. Maps are originals and were generated using the Excel function Map chart, with Bing technology © GeoNames, HERE, MSFT, Microsoft, NavInfo, Wikipedia.

We analyzed all five codons with currently known PY-predictive mutations (Fig 1A) in 512 samples from twelve populations, including mosquitoes from the native home range (China and Thailand), established areas (La Reunion Island) and newly invaded regions (i.e. Mexico, Greece and Italy) (Table 1) [9]. We identified mutations in all tested samples. As summarized in Fig 2, Table 2 and Table 3, the distribution and frequency of *kdr* mutations was variable across populations. In domain I, mutations at codon 410 were detected in different allelic variants besides the previously-identified V410L [31]. V410L was detected in mosquitoes from Thailand, Mexico and Italy, always in heterozygosity and with frequencies ranging from 0.57% (Mexico) to 2.94% (Italy). In mosquitoes collected in Italy, we also detected changes from V to either A or G both in heterozygosity and homozygosity and with frequency ranging between 4.4 and 5.9% (Table 3). In domain II, at codon 989, we detect the previously-identified S989P variant [20] in heterozygosity in one mosquito from Thailand and a change to Y in southern Mexico and Italy with frequencies ranging from 0.57 to 3.71%, respectively. At codon 1011, the PY-linked mutation I1011V was found in heterozygosity in one Italian mosquito (Table 3). At position 1016, we found both the V1016G and the V1016I mutations, which are associated with PY-resistance, in China, Italy and la Reunion Island at low frequency (<5.56%) and always in heterozygosity.

**Table 1:** List of *Ae*. *albopictus* samples from native, established and invasive populations used in this study.

Region	Collection site	Status	Samples	No. Mosquitoes	Collection year
Asia	China, Guangzhou	Native	CG	40	2017
	Thailand, Uthai Thani province		TU	30	2011
	Thailand, Samut Sakhon province		TS	30	2018
Indian Ocean	La Réunion Island, Saint Pierre	Established	FR	23	2016
Central America	Mexico, Chiapas	Invasive	MC	183	2016
Mediterranean area	Greece, Athens		GA	30	2014
	Northern Italy, Modena		IM	30	2011
	Northern Italy, Comacchio		IO	30	2011
	Northern Italy, Arco		IA	36	2012
	Northern Italy, Turin		IT	30	2013
	Southern Italy, Foggia		IF	30	2011
	Southern Italy, Cosenza		IC	20	2018

**Table 2:** Allele frequencies results of *kdr* mutations in *Ae*. *albopictus* worldwide populations. For each codon, the first column on the left shows the wild-type amino acid.

		Domain I				Domain II									Domain III				
	Codon	410				989			1011			1016			1534				
Sample	AA	V	L	A	G	S	P	Y	I	M	V	V	G	I	F	C	L	W	S
**CG**		100	0	0	0	100	0	0	100	0	0	97.44	2.56	0	16.67	8.33	4.17	4.17	66.66
**TU**		97.92	2.08	0	0	98.33	1.67	0	100	0	0	100	0	0	100	0	0	0	0
**TS**		100	0	0	0	100	0	0	100	0	0	100	0	0	100	0	0	0	0
**FR**		100	0	0	0	100	0	0	100	0	0	97.62	2.38	0	100	0	0	0	0
**MC**		99.43	0.57	0	0	99.43	0	0.57	100	0	0	100	0	0	97.99	0	2.01	0	0
**GA**		100	0	0	0	100	0	0	100	0	0	100	0	0	50	50	0	0	0
**IA**		95.59	0	4.41	0	100	0	0	100	0	0	100	0	0	100	0	0	0	0
**IT**		98.21	1.79	0	0	100	0	0	100	0	0	96.67	3.33	0	100	0	0	0	0
**IF**		100	0	0	0	100	0	0	100	0	0	98	2	0	100	0	0	0	0
**IO**		100	0	0	0	96.29	0	3.71	100	0	0	100	0	0	100	0	0	0	0
**IM**		100	0	0	0	100	0	0	97.22	0	2.78	94.44	5.56	0	100	0	0	0	0
**IC**		91.18	2.94	0	5.88	100	0	0	100	0	0	97.5	0	2.5	100	0	0	0	0

**Table 3:** Allele genotyping results of *kdr* mutations in *Ae*. *albopictus* worldwide populations. For each codon, the first column on the left shows the homozygote wild-type genotype. Only genotypes with frequencies different from 0 are shown.

		Domain I					Domain II									Domain III							
		
	Codon	410					989			1011		1016				1543							
Sample	Genotype	VV	VL	VA	AA	VG	SS	SP	SY	II	IV	VV	VI	VG	FF		FC	CC	FL	LL	WW	FS	SS
**CG**		100	0	0	0	0	100	0	0	100	0	94.87	0	5.13	12.5		0	8.33	0	4.17	4.17	8.33	62.5
**TU**		95.83	4.17	0	0	0	96.67	3.33	0	100	0	100	0	0	100		0	0	0	0	0	0	0
**TS**		100	0	0	0	0	100	0	0	100	0	100	0	0	100		0	0	0	0	0	0	0
**FR**		100	0	0	0	0	100	0	0	100	0	95.24	0	4.76	100		0	0	0	0	0	0	0
**MC**		98.85	1.15	0	0	0	98.85	0	1.15	100	0	100	0	0	95.98		0	0	0	4.02	0	0	0
**GA**		100	0	0	0	0	100	0	0	100	0	100	0	0	25		50	25	0	0	0	0	0
**IA**		94.12	0	2.94	2.94	0	100	0	0	100	0	100	0	0	100		0	0	0	0	0	0	0
**IT**		96.43	3.57	0	0	0	100	0	0	100	0	93.33	0	6.67	100		0	0	0	0	0	0	0
**IF**		100	0	0	0	0	100	0	0	100	0	96	0	4	100		0	0	0	0	0	0	0
**IO**		100	0	0	0	0	92.59	0	7.41	100	0	100	0	0	100		0	0	0	0	0	0	0
**IM**		100	0	0	0	0	100	0	0	94.44	0	88.89	0	11.11	100		0	0	0	0	0	0	0
**IC**		82.35	5.88	0	0	11.77	100	0	0	100	0	95	5	0	100		0	0	0	0	0	0	0

Finally, at domain III we confirmed the presence of three variants at codon 1534, as previously shown [35], and we identified a new mutation to W, in China (Table 2). Alarmingly, the F1534C mutation, which is predictive of the resistant phenotype [37], was found in 50% of the tested mosquitoes from Greece (Table 2).

Among all the samples analyzed, instances of double mutants were rare. We identified the double mutation S989Y-F1534L in mosquitoes from Mexico and China.

### Temporal variation in the frequency of *kdr* mutations

Temporal variation in the frequency of *kdr* mutations was analyzed comparing data from our collections, which were sampled between 2011 to 2018, and data from previous studies [35,37,38] (Table 4). The most drastic increase in allelic frequency was observed for mutations at position 1534. The F1534C mutation increased 40% in Greece (Athens) in three years and the F1534S mutation increased 60% in China (Guanghzou) in five years. Significant changes in allele frequency were also observed for V1016G, which increased about 30% in mosquitoes from the Emilia Romagna region in northern Italy (Modena sample). The increase in the frequency of V1016G was not associated with changes in the frequency of the linked S989Y mutation, which was only recorded in 2011 in Emilia Romagna, but in a different population (Comacchio sample). Mutations at codons 410, 989 and 1011 were studied for the first time here.

**Table 4:** Temporal variation in PY-predictive mutations in *Ae*. *albopictus* mosquitoes. Boxes are empty when data are not available.

		Domain II			Domain III				
Codon		1016			1534				
Site	Year	V	G	I	F	C	L	W	S
**China, Guangzhou**	2014*				63.5	0	24.5	0	9.5
	**2017**	97.44	2.56	0	16.67	8.33	4.17	4.17	66.66
**La Réunion Island**	2012*				100	0	0		0
	**2016**	97.62	2.38	0	100	0	0	0	0
**Northern Italy, Arco**	2011*				99	0	1		
	**2012**	100	0	0	100	0	0	0	0
**Northern Italy, Turin**	**2013**	96.67	3.33	0	100	0	0	0	0
	2017**	100	0	0					
**Northern Italy, Modena**	**2011**	94.44	5.56	0	100	0	0	0	0
	2016**	64	36	0	100	0			
**Greece, Athens**	2013*				75.8	24.2	0		0
	**2014**	100	0	0	50	50	0	0	0
	2016**	100	0		34	66			

*data from Xu et al., 2016 [35]

**data from Pichler et al., 2018 [22].

### Origin of *kdr* haplotypes

**Fig 3:**
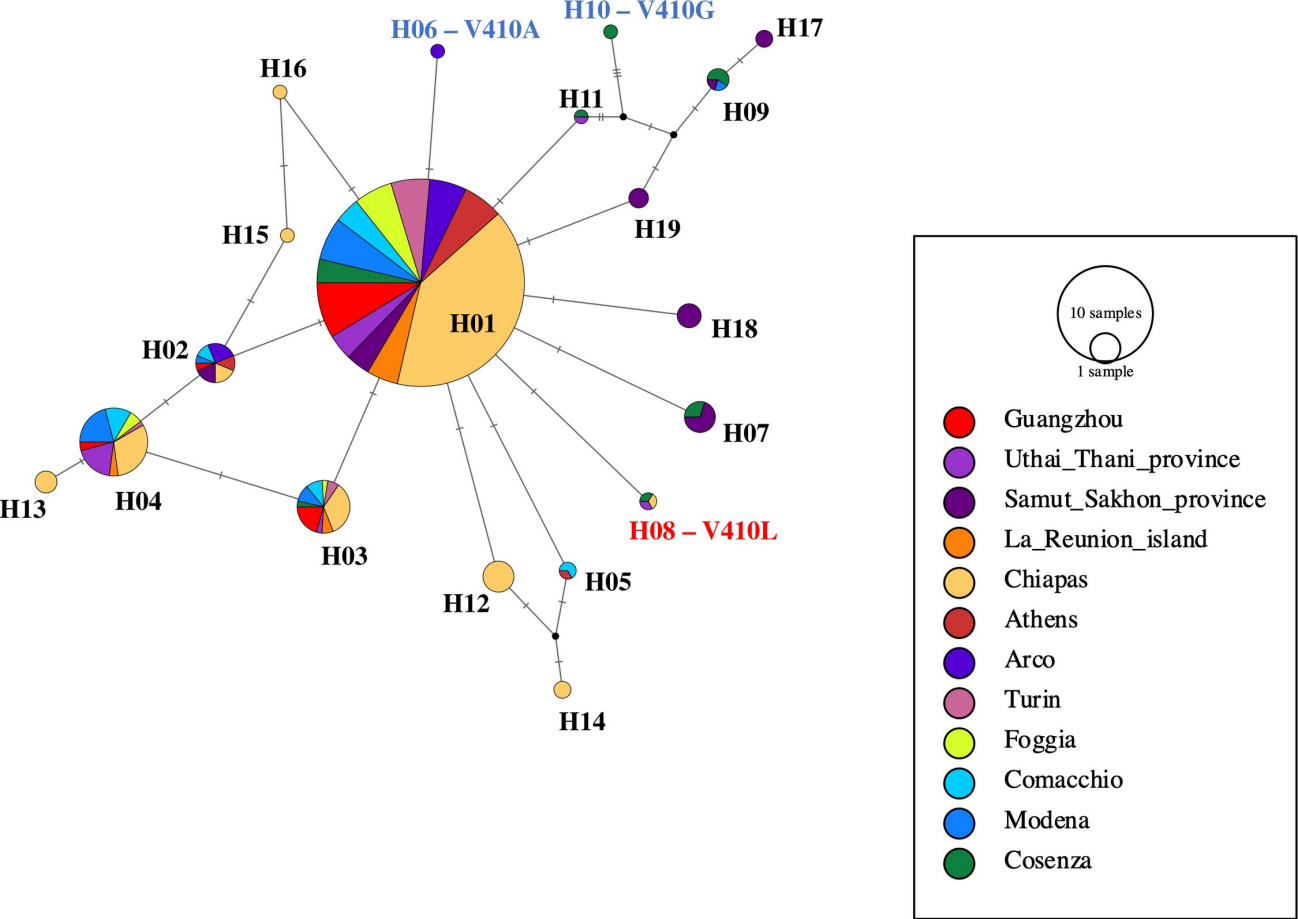
Genealogical network among haplotypes in Domain I. Wild-type haplotypes are in black. Haplotypes with *kdr* mutations predictive of the resistance phenotype are in red. Haplotypes with alternative *kdr* mutations are in blue.

In domain I, a total of 15 polymorphic sites were identified. The haplotype of 142 individuals, which were heterozygotes at more than one site, was predicted with PHASE, leading to a total of 598 sequences distributed into 19 haplotypes (S1 Table). The genealogical network among haplotypes shows the mutation V410L on haplotype H8, which is shared among mosquitoes from Italy, Mexico and Thailand. Two distinct haplotypes, H6 and H10, bear the V410A and V410G mutations in mosquitoes from Arco and Cosenza, respectively. While H8 and H6 derive from single mutational steps from the same ancestor H1; H10 is the results of several mutation steps from H11, a haplotype shared between mosquitoes from Thailand and Italy (Fig 3).

At domain II, polymorphic sites were distributed in both exons 20 and 21 and the intron in between, leading to a total of 770 sequences distributed into 74 haplotypes (S1B Table). *kdr* mutations at positions 989, 1011 and 1016 were distributed in different haplotypes, indicating independent origins (S2 Fig). Mutations at position 989 were found in four population-specific haplotypes (i.e. H56, H58, H62, H74). The 989Y variant is distributed into three haplotypes: H56 and H58, both in mosquitoes from Comacchio (Italy), whereas H62 detected in mosquitoes from Mexico, derives directly from H01. The 989P variant is found on H74 in mosquitoes from Thailand. The I1011V mutation was found on the H55 haplotype, which derives from several mutation step from the ancestor H13. The V1016G mutation was found in three haplotypes (H8, H10, H59). While haplotype H10 was shared among mosquitoes from China, La Reunion Island and Italy, the other two haplotypes were uniquely found in China or Italy. H10 derives from one mutation step from H07; H08 derives from H09; whereas H59 derives from H49, which is the results of mutational steps from both H09 and H07.

At domain III, polymorphic sites were analyzed in exon 29 leading to a total of 763 sequences distributed in 45 haplotypes (S1C Table). Interestingly, while mosquitoes from Greece having the F1534C mutation share the same H10 haplotype, in mosquitoes from China the F1534C/S/L/W mutations were distributed into different haplotypes, suggesting independent emergence of mutations at position 1534 (S3 Fig). The mutation F1534C is distributed into two haplotypes (H10, H12), which both derive from the same ancestor H11, after numerous mutation steps. F1534S is distributed across seven haplotypes (H01, H02, H03, H04, H05, H06, H08), which derive from the ancestor H11. The F1534L is present in four haplotypes (H07, H40, H42, H44) derived from H11.

### Genetic connectivity of Mexican mosquitoes

**Fig 4:**
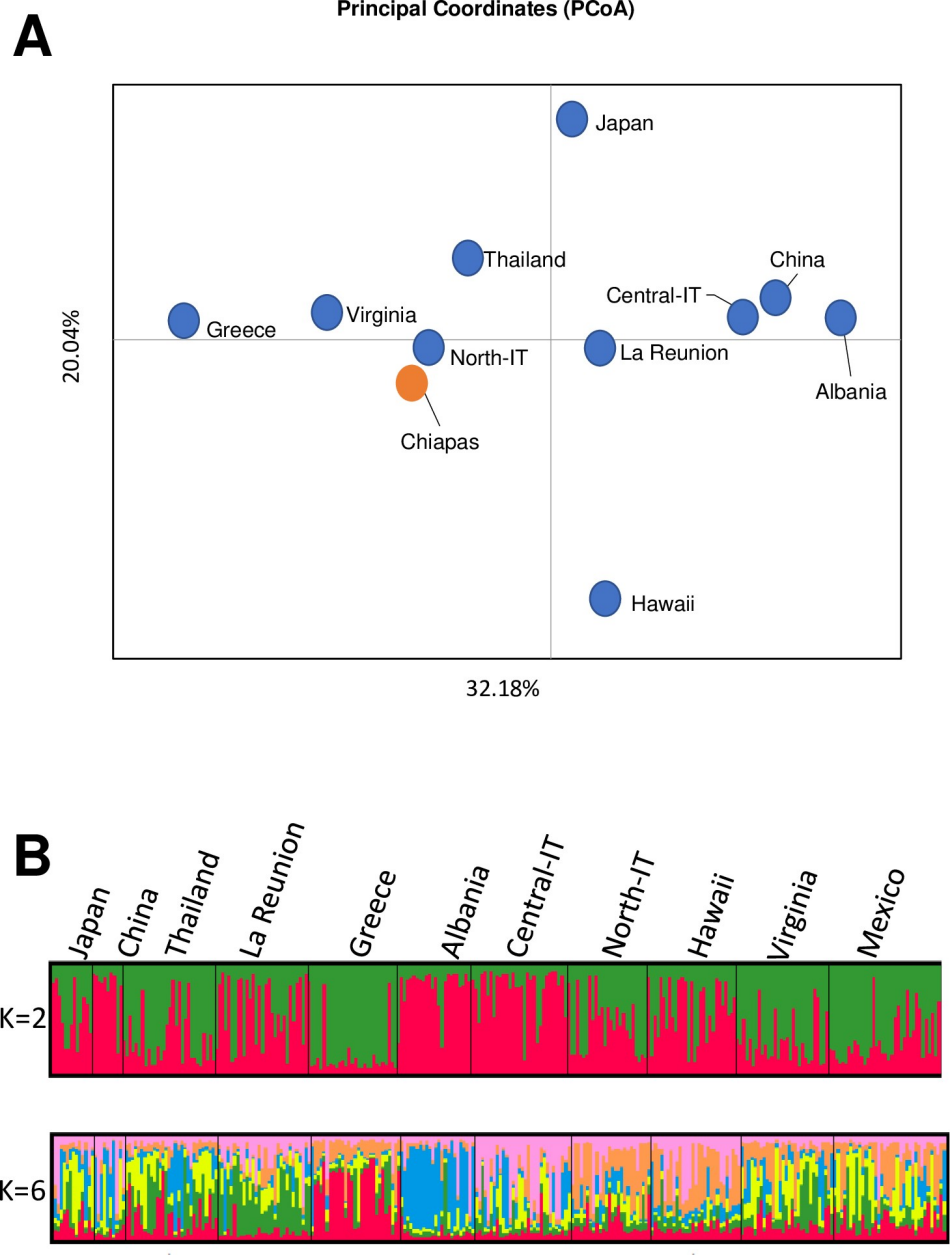
Genetic structure of *Ae*. *albopictus* populations as revealed by microsatellite markers. A) Principal coordinate analysis (PCOA) based on the calculated Fst values. Mosquitoes from Chiapas are shown by an orange dot. B) Graphical representation of the Bayesian cluster analysis using STRUCTURE with K = 2 and K = 6.

Among the analyzed regions, mosquitoes from Mexico showed *kdr* mutations at position 410, 989 and 1534 (Tables 2 and 3). *Aedes albopictus* was first found in Mexico in the northern state of Tamaulipas, which borders Texas, in 1988 [9], but it was recorded for the first time in the southern state of Chiapas only in 2002 [40]. Because of these characteristics, mosquitoes from Chiapas are ideal to test whether *kdr* mutations are likely to arise locally or be imported. Thus, we analyzed 36 mosquitoes from Chiapas using five microsatellite loci and integrated results with previously-analyzed world-wide samples [41]. Levels of genetic variability in mosquitoes from Chiapas were comparable to what detected in previously-analyzed worldwide mosquitoes [11]. For instance, the number of effective alleles/locus ranged between 1.687 (Hawaii) and 3.841 (Thailand), with a value of 2.307 for Chiapas; the observed heterozygosity ranged between 0.283 (Hawaii) and 0.520 (La Reunion Island), with a value of 0.472 for Chiapas (S2 Table). Despite mosquitoes from Chiapas having a similar number of effective alleles as other tested populations, they also showed private alleles at different loci (i.e. Aealbmic2; Aealbmic3; Aealbmic17; Aealbmic9), a mark of genetic distinctiveness (S2 Table). Fst values of mosquitoes from Chiapas were lower in comparison to those of mosquitoes from other invasive populations such as northern Italy, Virginia and Greece, suggesting invasion into southern Mexico is a secondary invasion event, possibly from different regions (Fig 4A) [42]. We further examined genetic structuring of individuals using the Bayesian method implemented in STRUCTURE [43]. Running STRUCTURE, followed by the Evanno method (ΔK) [43], resulted into two peaks at K = 2 and K = 6, which suggests two scenarios of genetic clustering for the tested populations. Individual mosquitoes from the examined populations were assigned to each cluster with a certain probability value for the two scenarios of 2 and 6 clusters (S4 Table). In both scenarios, extensive genetic admixture among *Ae*. *albopictus* geographic samples was evident as previously observed [10,11] (Fig 4B). Regarding mosquitoes from Chiapas, in the two-cluster model, they clustered with Virginia, Greece and north Italy. In the six-cluster model, the Mexican mosquito population appeared divided into subgroups showing similarities with mosquitoes from either Virginia, Greece or north Italy, supporting the hypothesis of secondary invasions from multiple regions.

## Discussion

In this study we used sequencing data of the three domains of the *vgsc* gene to test the distribution pattern of *kdr* mutations predictive of the resistance phenotype in native, established and invasive populations of *Ae*. *albopictus*, including samples collected in different years. To test for the origin of *kdr* mutations, we derived haplotype networks and, focusing on samples from Chiapas for which we have detailed historical data, we tested whether the origin of Mexican mosquitoes as derived by microsatellite data correlates with their *kdr* haplotypes.

An increase in the geographical distribution of PY-predictive resistance mutations was observed. Additionally, we detected mutations at all currently-known *kdr* codons, including position 410 so far analyzed only in *Ae*. *aegypti* (Fig 1B and 1C) [20,31]. Resistance alleles were mostly found in a heterozygous state and instances of double mutants were rare, suggesting resistance to PY is still at its emergence state in most analyzed *Ae*. *albopictus* world-wide samples. However, an alarming increase in the frequency of *kdr* mutations was detected in China, Italy and Greece over a five-year span. In China and Greece, we observed an increase of mutations at position 1534, albeit of different alleles. In Italy, we observed mutations at position 1016 as early as 2011 and their low, but steady increase in the following years, up to 2018. Our results indicate that mutations at position 1534 and 1016, which are both highly predictive of the resistance phenotype [35,37], can evolve independently in *Ae*. *albopictus*. Multiple origins of *kdr* mutations is also supported by haplotype network analysis, which showed at least three, four and six independent origins of *kdr* alleles in domain I, II and III of the *vgsc* gene, respectively. Multiple origins of *kdr* mutations is a common phenomenon in Culicinae mosquitoes, having already been detected in *Anopheles gambiae*, *Anopheles sinensis and Ae*. *aegypti* [44–47]. In support of the hypothesis of independent and local origin of *kdr* mutations, mosquitoes from Chiapas showed a pattern of *kdr* mutations different than that of mosquitoes from Virginia, Greece and northern Italy to which they were most genetically-close. Finally, both at codons 1016 and 1534 and codons 410 and 989, we observed additional variants than the ones previously-liked to PY resistance [20]. While we recognize the importance of conducting resistance bioassays to confirm the association between these novel alleles and phenotypic resistance, logistics constraints prevented us to perform larvae collections and phenotypic assays for this study. Albeit recognizing this limitation, we emphasize the importance of our results. We provide the most-up-to-date survey on the widespread distribution of PY-predictive mutations (i.e. F1534C/S; V1016G) in *Ae*. *albopictus* by combining *kdr* genotyping on current and historical samples with published data. Considering the recent dispersal of *Ae*. *albopictus* out of its native range, which resulted in the overlay of mosquitoes from different origins in newly colonized areas, and the current spatial heterogeneity in the distribution of PY resistance mutations [10,11,21,20,35,37–38], adding the temporal scale to the analyses of *kdr* mutations is important for motoring the emergence and identifying the speed of evolution.

Overall, our results show that the frequency of *kdr* mutations predictive of the resistance phenotype is low in most analyzed samples, but drastically on the rise in Italy, Greece and China, calling for the development of countermeasures. Additionally, our data support the hypothesis that on site emergence of resistance has contributed more than spread of resistance through mosquito migration/invasions to the observed patterns of *kdr* allele distribution, emphasizing the importance of local surveillance programs and resistance management.

## Material and methods

### Mosquito samples and DNA extraction

This study used *Ae*. *albopictus* samples collected in the field as adults, which were preserved in 70%–100% ethanol until DNA extraction. Table 1 lists sampling sites and number of samples per location. Briefly, from the native home range, we analyzed mosquitoes collected in 2017 in Guangzhou (China), three years after our first survey on *kdr* mutations [35], and samples collected in the Thai western provinces of Uthai Thani and Samut Sakhon in 2011 and 2018, respectively. Additionally, we analyzed a 2016 collection from La Reunion Island, an old colonized area where a survey on 2012-collected mosquitoes detect no *kdr* mutations [9,35] and various invasive populations from Europe and Mexico sampled between 2011 and 2018.

Genomic DNA was extracted from a single leg of each mosquito. For each specimen, mosquito species identity was confirmed by amplification of the ribosomal internal transcribed spacer ITS2 using species-specific primers [48].

### *Kdr* genotyping

Polymorphism of the *vgsc* gene was studied in a total of 512 *Ae*. *albopictus* mosquitoes using three set of primers. We amplified exon 10 in domain I with primers aegSCF10 (GTGTTACGATCAGCTGGACC) and aegSCR10 (AAGCGCTTCTTCCTCGGC); the region containing exons 20 and 21 in domain II and exon 29 in domain III with the previously-described set of primers aegSCF20/aegSCR21 and aegSCF7/aegSCR8, respectively [48]. Domain I includes position 410; domain II encompasses positions 989, 1011 and 1016 and domain III incudes position 1534 in which mutations predictive of the resistance phenotype have been characterized in both *Ae*. *aegypti* and *Ae*. *albopictus* (Fig 1) [20,31–39,49]. PCR mixes of 30 μl final volume were set up containing 15 μl Master Mix (Thermo Fisher Scientific Inc., Waltham, MA, USA), 2 μl of template DNA and 1mM of each primer. PCR reactions were run in an Eppendorf Thermal Cycler. Amplification reactions were set up with an initial step at 95°C for 10 min, followed by 40 cycles at 95°C for 30 sec, annealing temperature depending on the tested primer set for 30 sec, 72°C for 30–45 sec depending on the length of the expected sequence and a final extension step of 72°C for 7 min.

PCR products were purified using the Exo-SAP reagents (Affymetrix) according to the manufacturer’s protocol and directly sequenced using the service from Macrogen Europe (Spain) service.

The chromatogram of each sequence was visually inspected by using Unipro UGENE (http://ugene.net/). Heterozygotes were called in case of peaks with equal amplitude. Cases of ambiguity were confirmed by cloning, and then sequencing, PCR-amplified DNA fragments from a subset of 20 individuals.

### *Kdr* haplotypes

DNA sequences of each domain were aligned using Unipro UGENE (http://ugene.net/). DNA polymorphism (i.e. number of segregating sites, number and diversity of haplotypes, nucleotide diversity and Tajima’s D) was analyzed using DnaSPv5 [50]. The Phase 2.1 program within DnaSPv5 was used to reconstruct haplotypes from the genotypic data of mosquitoes which were heterozygotes at more than one site. These haplotypes were confirmed by cloning, and then sequencing, of DNA fragments from a subset of 20 individuals. The pCR2.1 TOPO TA cloning vector strategy (Invitrogen, Carlsbad, CA, USA) was used for cloning. The haplotypes in this study were deposited in the GenBank database (Accession numbers MN954411-MN954473, MN956909-MN956985).

The software TCS v1.21 [51] was used to estimate the genealogical relationships among haplotypes. The program implements statistical parsimony to build a network of haplotypes, drawn to scale based on their frequencies, and estimate the number of mutational steps with a connection probability threshold of 95% between pairs of haplotypes [51]. The program PopART was used to visualize TCS-based haplotype networks [52].

### Microsatellite analyses

Samples from Chiapas (Mexico) were screened for five previously-characterized microsatellite loci using the same procedure as described [41]. These loci (i.e. Aealbmic2, Aealbmic3, Aealbmic5, Aealbmic9, Aealbmic17) were chosen because they are spread across the *Ae*. *albopictus* genome and showed to be highly polymorphic [11].

Genetic variation in Mexican samples was estimated calculating the average number of alleles (n_a_), the number of private alleles (n_p_) and their frequency (A_p_) in GenAlEx6.5 [53]. The genetic relationships between Mexican samples and previously-analysed worldwide samples were examined using STRUCTURE V 2.3.2 [11], setting 500,000 burn-in iterations and 500,000 MCMC. Allele frequency were assumed to be correlated. The number of clusters (K) was set between 1 and 11 (i.e. the number of populations tested) and ten independent runs were set for each K analysis. Results were analyzed in Structure Harvester [54] and plotted using the software CLUMMP 1.1.2 and Distruct 1.1 [55,56].

## Supporting information

S1 Table Haplotypes identified at Domain I (A), Domain II (B) and Domain III (C)
(XLSX)

S2 Table Genetic variability estimates for *Ae*. *albopictus* geographic samples at five microsatellite loci. Sample size is shown in parenthesis next to the name of the sample. Na = number of alleles, Ne = number of effective alleles, Ho = observed heterozygosity, He = Expected Heterozigosity, uHe = Unbiased expected heterozygosity, F = Fixation, Index, I = Shannon Information Index; Pa = number of private alleles; SE = Standard Error.
(DOCX)

S3 Table Pairwise Population Fst values. (DOCX)

S4 Table Membership coefficient value for each cluster of the tested *Ae*. *albopictus* populations with K = 2 (A) and K = 6 (B).
(DOCX)

S1 Fig Cluster analysis of microsatellite genotyping. Delta K values were calculated using STRUCTURE. Data presents relation between Delta L and number of clusters (k). The highest Delta K values correspond to k = 2 and K = 6.
(TIF)

S2 Fig Genealogical network among haplotypes in Domain II. Wild-type haplotypes are in black. Haplotypes with *kdr* mutations predictive of the resistance phenotype are in red. Haplotypes with alternative *kdr* mutations are in blue.
(TIF)

S3 Fig Genealogical network among haplotypes in Domain III. Wild-type haplotypes are in black. Haplotypes with *kdr* mutations predictive of the resistance phenotype are in red. Haplotypes with alternative *kdr* mutations are in blue.
(TIF)
