# Supplementary material for: Tracing temporal and geographic distribution of resistance to pyrethroids in the arboviral vector Aedes albopictus
Source: PLoS Negl Trop Dis. 2020 Jun 22;14(6):e0008350. doi: 10.1371/journal.pntd.0008350 (PMC7332087; doi:10.1371/journal.pntd.0008350)
Supplement: S2 Table — Sample size is shown in parenthesis next to the name of the sample. Na = number of alleles, Ne = number of effective alleles, Ho = observed heterozygosity, He = Expected Heterozigosity, uHe = Unbiased expected heterozygosity, F = Fixation, Index, I = Shannon Information Index; Pa = number of private alleles; SE = Standard Error. (DOCX) [file pntd.0008350.s002.docx]

**Supplemental Table 2.** Genetic variability estimates for *Ae. albopictus* geographic samples at five microsatellite loci. Sample size is shown in parenthesis next to the name of the sample. Na= number of alleles, Ne=number of effective alleles, Ho= observed heterozygosity, He= Expected Heterozigosity, uHe= Unbiased expected heterozygosity, F= Fixation Index, I= Shannon Information Index; Pa=number of private alleles; SE= standard Error

|  |  | **Na** | **Ne** | **Ho** | **He** | **uHe** | **F** | **I** | **Pa** |
| --- | --- | --- | --- | --- | --- | --- | --- | --- | --- |
| **Japan (13)** | Mean | 5.800 | 2.617 | 0.500 | 0.527 | 0.548 | 0.045 | 1.133 | 0.400 |
|  | SE | 1.625 | 0.483 | 0.130 | 0.134 | 0.139 | 0.074 | 0.308 | 0.400 |
| **China (10)** | Mean | 4.000 | 2.554 | 0.424 | 0.537 | 0.567 | 0.290 | 1.003 | 0.400 |
|  | SE | 0.837 | 0.566 | 0.138 | 0.084 | 0.088 | 0.225 | 0.206 | 0.245 |
| **Thailand (30)** | Mean | 6.800 | 3.841 | 0.480 | 0.594 | 0.605 | 0.194 | 1.306 | 0.400 |
|  | SE | 2.131 | 1.063 | 0.127 | 0.156 | 0.158 | 0.015 | 0.384 | 0.245 |
| **La Reunion (30)** | Mean | 5.200 | 3.717 | 0.520 | 0.557 | 0.567 | 0.041 | 1.157 | 0.400 |
|  | SE | 1.881 | 1.175 | 0.136 | 0.157 | 0.160 | 0.058 | 0.394 | 0.400 |
| **Greece (29)** | Mean | 4.600 | 2.556 | 0.372 | 0.502 | 0.511 | 0.262 | 0.976 | 0.800 |
|  | SE | 1.166 | 0.547 | 0.111 | 0.135 | 0.138 | 0.099 | 0.268 | 0.800 |
| **Albania (24)** | Mean | 4.200 | 2.404 | 0.369 | 0.489 | 0.499 | 0.364 | 0.951 | 0.400 |
|  | SE | 0.860 | 0.629 | 0.112 | 0.102 | 0.104 | 0.163 | 0.221 | 0.245 |
| **Central-IT (31)** | Mean | 5.800 | 2.948 | 0.419 | 0.532 | 0.541 | 0.205 | 1.147 | 0.200 |
|  | SE | 1.428 | 0.721 | 0.128 | 0.145 | 0.147 | 0.101 | 0.332 | 0.200 |
| **North-IT (26)** | Mean | 3.200 | 2.261 | 0.446 | 0.471 | 0.481 | 0.022 | 0.815 | 0.200 |
|  | SE | 0.860 | 0.434 | 0.120 | 0.124 | 0.127 | 0.122 | 0.242 | 0.200 |
| **Hawaii (29)** | Mean | 2.800 | 1.687 | 0.283 | 0.345 | 0.351 | 0.289 | 0.581 | 0 |
|  | SE | 0.374 | 0.246 | 0.101 | 0.107 | 0.109 | 0.189 | 0.166 | 0 |
| **Virginia (30)** | Mean | 5.800 | 2.821 | 0.572 | 0.531 | 0.540 | -0.085 | 1.113 | 0.600 |
|  | SE | 1.715 | 0.631 | 0.151 | 0.141 | 0.143 | 0.056 | 0.326 | 0.400 |
| **Mexico (36)** | Mean | 6.200 | 2.307 | 0.472 | 0.548 | 0.556 | 0.152 | 1.094 | 1.800 |
|  | SE | 0.735 | 0.203 | 0.067 | 0.052 | 0.053 | 0.065 | 0.079 | 1.068 |
