# Supplementary material for: Tracing temporal and geographic distribution of resistance to pyrethroids in the arboviral vector Aedes albopictus
Source: PLoS Negl Trop Dis. 2020 Jun 22;14(6):e0008350. doi: 10.1371/journal.pntd.0008350 (PMC7332087; doi:10.1371/journal.pntd.0008350)
Supplement: S3 Table — (DOCX) [file pntd.0008350.s003.docx]

**Supplemental Table 3.** Pairwise population Fst values.

|  | **1** | **2** | **3** | **4** | **5** | **6** | **7** | **8** | **9** | **10** | **11** |
| --- | --- | --- | --- | --- | --- | --- | --- | --- | --- | --- | --- |
| **1-Japan** | 0.000 |  |  |  |  |  |  |  |  |  |  |
| **2-China** | 0.054 | 0.000 |  |  |  |  |  |  |  |  |  |
| **3-Thailand** | 0.039 | 0.069 | 0.000 |  |  |  |  |  |  |  |  |
| **4-La Reunion Island** | 0.070 | 0.065 | 0.031 | 0.000 |  |  |  |  |  |  |  |
| **5-Greece** | 0.084 | 0.112 | 0.036 | 0.076 | 0.000 |  |  |  |  |  |  |
| **6-Albania** | 0.073 | 0.043 | 0.061 | 0.054 | 0.121 | 0.000 |  |  |  |  |  |
| **7-Central-IT** | 0.050 | 0.045 | 0.059 | 0.040 | 0.103 | 0.059 | 0.000 |  |  |  |  |
| **8-North-IT** | 0.065 | 0.070 | 0.048 | 0.055 | 0.060 | 0.090 | 0.066 | 0.000 |  |  |  |
| **9-Hawaii** | 0.118 | 0.082 | 0.084 | 0.068 | 0.101 | 0.083 | 0.074 | 0.070 | 0.000 |  |  |
| **10-Virginia** | 0.062 | 0.084 | 0.032 | 0.069 | 0.038 | 0.093 | 0.093 | 0.036 | 0.085 | 0.000 |  |
| **11-Mexico** | 0.071 | 0.065 | 0.046 | 0.055 | 0.039 | 0.087 | 0.065 | 0.033 | 0.062 | 0.038 | 0.000 |
