# Supplementary material for: Tracing temporal and geographic distribution of resistance to pyrethroids in the arboviral vector Aedes albopictus
Source: PLoS Negl Trop Dis. 2020 Jun 22;14(6):e0008350. doi: 10.1371/journal.pntd.0008350 (PMC7332087; doi:10.1371/journal.pntd.0008350)
Supplement: S4 Table — Membership coefficient value for each cluster of the tested Ae. albopictus populations with K = 2 (A) and K = 6 (B). (DOCX) [file pntd.0008350.s004.docx]

| **A** | | |
| --- | --- | --- |
| **Pop** | **K1** | **K2** |
| Nagasaki | 0.4502 | 0.5498 |
| Xiamen | 0.1922 | 0.8078 |
| BanRai | 0.6785 | 0.3215 |
| StPierre | 0.4084 | 0.5916 |
| Athens | 0.8273 | 0.1727 |
| Tirana | 0.1858 | 0.8142 |
| Cesena | 0.2123 | 0.7877 |
| Brescia | 0.5624 | 0.4376 |
| Oahu | 0.3947 | 0.6053 |
| Manassas | 0.6989 | 0.3011 |
| Chiapas | 0.6793 | 0.3207 |

**Supplemental Table 4.** Membership coefficient value for each cluster of the tested *Ae. albopictus* populations with K=2 (A) and K=6 (B).

| **B** | | | | | | |
| --- | --- | --- | --- | --- | --- | --- |
| **Pop** | **K1** | **K2** | **K3** | **K4** | **K5** | **K6** |
| Nagasaki | 0.1536 | 0.1799 | 0.1714 | 0.1181 | 0.3014 | 0.756 |
| Xiamen | 0.521 | 0.3465 | 0.3053 | 0.612 | 0.1785 | 0.564 |
| BanRai | 0.2115 | 0.1614 | 0.607 | 0.2689 | 0.2160 | 0.815 |
| StPierre | 0.644 | 0.924 | 0.1936 | 0.4050 | 0.1450 | 0.996 |
| Athens | 0.4993 | 0.622 | 0.623 | 0.1126 | 0.1021 | 0.1616 |
| Tirana | 0.503 | 0.6226 | 0.1325 | 0.753 | 0.615 | 0.578 |
| Cesena | 0.649 | 0.1585 | 0.4267 | 0.995 | 0.1605 | 0.899 |
| Brescia | 0.1620 | 0.1021 | 0.1671 | 0.941 | 0.927 | 0.3820 |
| Oahu | 0.1064 | 0.1211 | 0.2635 | 0.977 | 0.557 | 0.3555 |
| Manassas | 0.1596 | 0.1502 | 0.526 | 0.1954 | 0.2368 | 0.2054 |
| Chiapas | 0.1693 | 0.1054 | 0.894 | 0.1457 | 0.2451 | 0.2451 |
