# Supplementary figures and images for: Tracing temporal and geographic distribution of resistance to pyrethroids in the arboviral vector Aedes albopictus
Source: PLoS Negl Trop Dis. 2020 Jun 22;14(6):e0008350. doi: 10.1371/journal.pntd.0008350 (PMC7332087; doi:10.1371/journal.pntd.0008350)

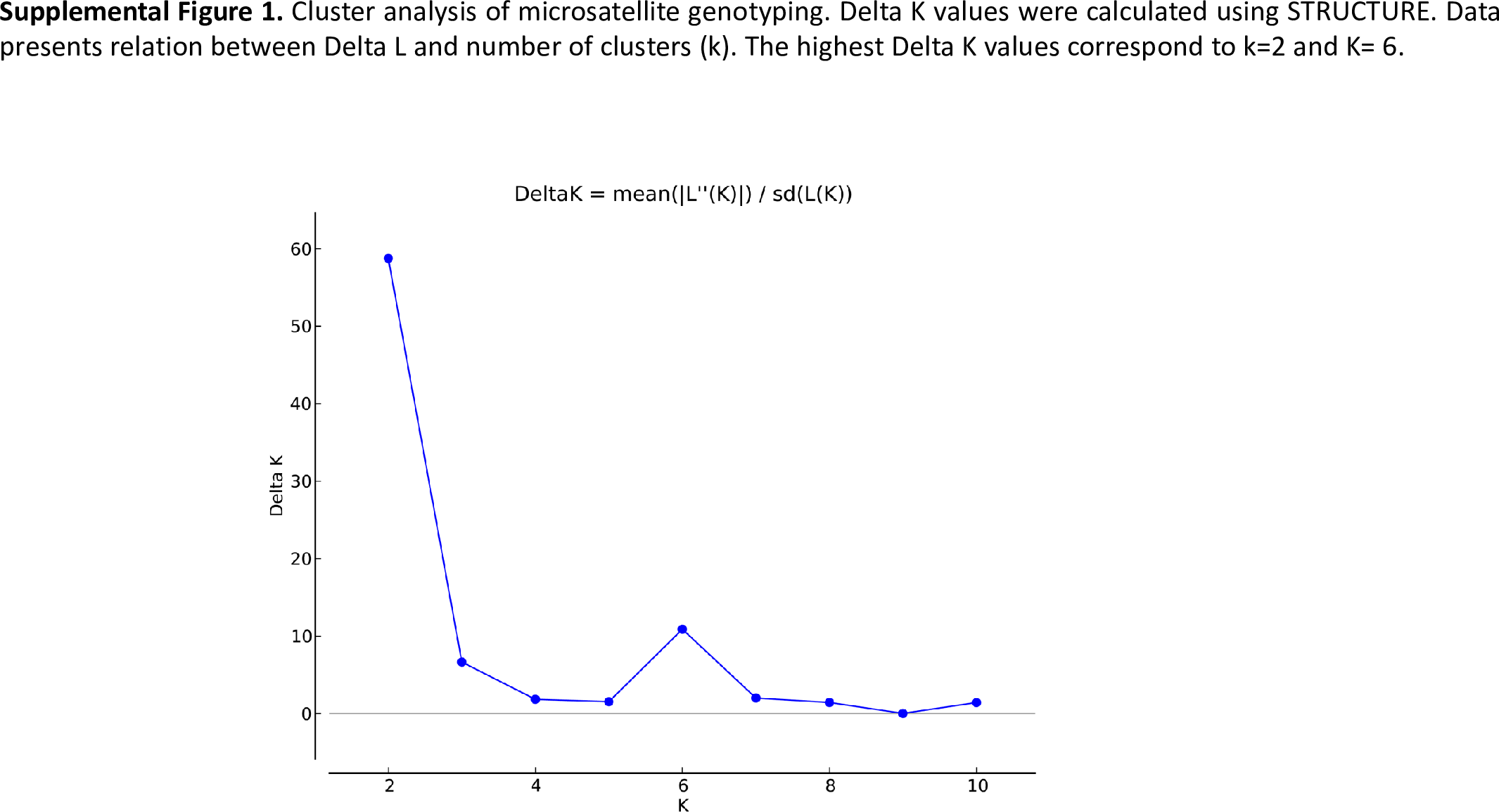

Supplement: S1 Fig — Delta K values were calculated using STRUCTURE. Data presents relation between Delta L and number of clusters (k). The highest Delta K values correspond to k = 2 and K = 6. (TIF) [file pntd.0008350.s005.tif]

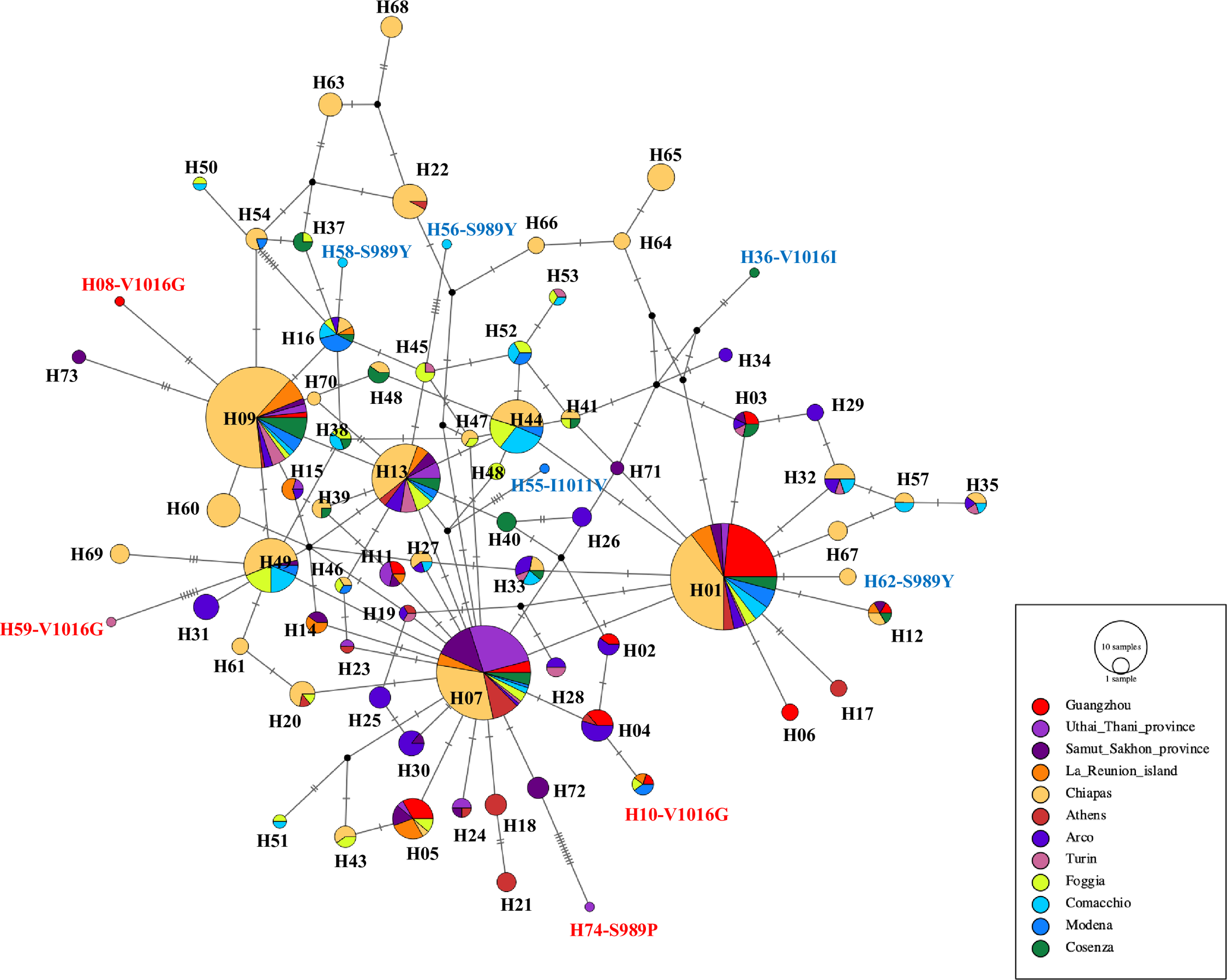

Supplement: S2 Fig — Wild-type haplotypes are in black. Haplotypes with kdr mutations predictive of the resistance phenotype are in red. Haplotypes with alternative kdr mutations are in blue. (TIF) [file pntd.0008350.s006.tif]

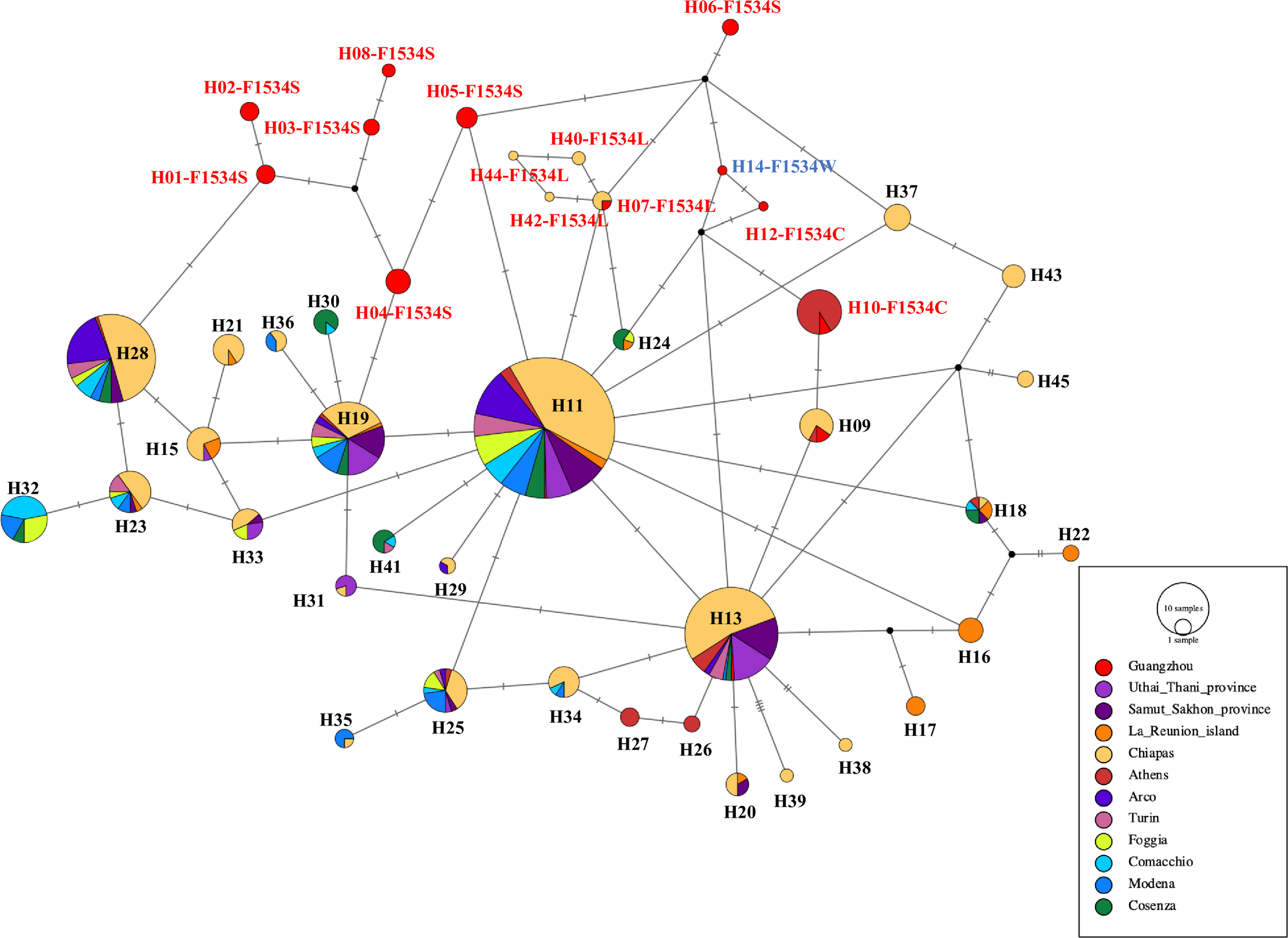

Supplement: S3 Fig — Wild-type haplotypes are in black. Haplotypes with kdr mutations predictive of the resistance phenotype are in red. Haplotypes with alternative kdr mutations are in blue. (TIF) [file pntd.0008350.s007.tif]
